# Edge- and Color–Texture-Aware Bag-of-Local-Features Model for Accurate and Interpretable Skin Lesion Diagnosis

**DOI:** 10.3390/diagnostics15151883

**Published:** 2025-07-27

**Authors:** Dichao Liu, Kenji Suzuki

**Affiliations:** Biomedical Artificial Intelligence Research Unit, Institute of Integrated Research (IIR), Institute of Science Tokyo, 4259 Nagatsuta-cho, Midori-ku, Yokohama 226-8503, Kanagawa, Japan

**Keywords:** diagnosis, biomedical imaging, computer-assisted intervention, handcrafted and deep feature fusion

## Abstract

**Background/Objectives**: Deep models have achieved remarkable progress in the diagnosis of skin lesions but face two significant drawbacks. First, they cannot effectively explain the basis of their predictions. Although attention visualization tools like Grad-CAM can create heatmaps using deep features, these features often have large receptive fields, resulting in poor spatial alignment with the input image. Second, the design of most deep models neglects interpretable traditional visual features inspired by clinical experience, such as color–texture and edge features. This study aims to propose a novel approach integrating deep learning with traditional visual features to handle these limitations. **Methods**: We introduce the edge- and color–texture-aware bag-of-local-features model (ECT-BoFM), which limits the receptive field of deep features to a small size and incorporates edge and color–texture information from traditional features. A non-rigid reconstruction strategy ensures that traditional features enhance rather than constrain the model’s performance. **Results**: Experiments on the ISIC 2018 and 2019 datasets demonstrated that ECT-BoFM yields precise heatmaps and achieves high diagnostic performance, outperforming state-of-the-art methods. Furthermore, training models using only a small number of the most predictive patches identified by ECT-BoFM achieved diagnostic performance comparable to that obtained using full images, demonstrating its efficiency in exploring key clues. **Conclusions**: ECT-BoFM successfully combines deep learning and traditional visual features, addressing the interpretability and diagnostic accuracy challenges of existing methods. ECT-BoFM provides an interpretable and accurate framework for skin lesion diagnosis, advancing the integration of AI in dermatological research and clinical applications.

## 1. Introduction

In recent years, the rising incidence of skin disease mortality has garnered public attention [1], and deep learning techniques such as ResNets [2] have become dominant computer-aided dermatologic diagnosis methods. However, despite their promising success, deep models are often regarded as “black boxes,” as their decision-making process is not always transparent or clinically interpretable. Given an image of a skin lesion, they can predict the lesion category but cannot explain the prediction’s basis. To address this issue, many previous studies have used attention visualization (AV) methods, such as class activation maps (CAMs) [3] and gradient-based class activation maps (Grad-CAM) [4], to explain the prediction basis of deep models in dermatologic diagnosis [5,6,7,8]. AV refers to generating a heatmap to highlight the regions in the input image leading to the model’s prediction. For example, Jiang et al. [6] added a CAM to some attention modules to generate a heatmap that identifies the lesion regions considered the decision basis. Wang et al. [5] utilized Grad-CAM as a part of an interpretable module to produce a heatmap for explaining the dermatologic diagnostic results.

However, many recent studies have pointed out that the heatmap yielded by existing AV methods is imprecise [9,10,11]. Existing AV methods usually employ a model’s deep layer to obtain a low-resolution activation map and up-sample it to create a heatmap of the same size as the input. This strategy requires the sampling results to be aligned with the input image spatially. However, the receptive field is very large in deep layers. For example, in ResNet50 [2], the receptive field of each pixel of a deep feature map can be even larger than the original input [12]. That is, in this case, from a theoretical point of view, each pixel of the deep activation maps may potentially sense any location on the input; thus it is not reasonable to use them to identify particular locations. From a practical point of view, deep activation maps somehow do correlate spatially with the input image. However, the underlying problem of too large receptive fields still causes many surface problems such as receptive field misalignment [9]. These problems have also been observed previously by other researchers [9,10,11].

Additionally, due to the large receptive field, most deep models are highly effective at capturing global features but struggle to capture fine-grained spatial details. However, for skin disease classification, these fine details are crucial. The differences between skin disease categories, such as melanoma and nevus, are often subtle and lie in fine-grained features like edge irregularity and color unevenness. While these diseases may appear similar in overall shape, it is these detailed characteristics that often determine whether a lesion is malignant or benign.

Another issue with today’s deep learning-based dermatologic diagnostics is the neglect of statistically-based traditional visual features derived from clinical experience, which are mathematically interpretable. For example, extracting edge information from a lesion and checking whether the pigment pattern shows a sharp, abrupt cutoff or a gradual, inconspicuous one is a crucial step in the clinical diagnostic process of a skin lesion [13,14]. Therefore, traditional computer-aided diagnostics often utilize edge information (EI) such as Sobel [15] as part of the descriptors [16]. Other examples include color–texture information (CTI), which also plays a crucial role in both clinical and traditional computer-aided diagnosis of skin lesions [13]. Although it is possible for a deep model to implicitly acquire features with similar functionality to traditional ones during feature extraction, it remains uncertain whether they actually do so. Additionally, understanding how deep models base decisions on these features is unattainable.

In response to the above issues, we revisited the dermatological image diagnosis process used before the rise in deep learning. In this traditional approach, handcrafted features are first extracted from small image patches and then statistically analyzed before being aggregated into image-level representations [17]. This method has two advantages: first, the extracted features from each patch are statistically interpretable; second, identifying the most influential patches helps pinpoint the visual cues contributing to the diagnosis. Inspired by this, we explore *whether modern deep learning systems can retain these advantages while maintaining their performance benefits.* This led us to bag-of-feature (BoF) models [18], which classify an image based on the occurrence of small local images, and we propose the edge- and color–texture-aware bag-of-local-features model (ECT-BoFM), which allows the BoF model to draw insights from traditional features without being constrained by them, thereby further enhancing performance.

**Figure 1:**
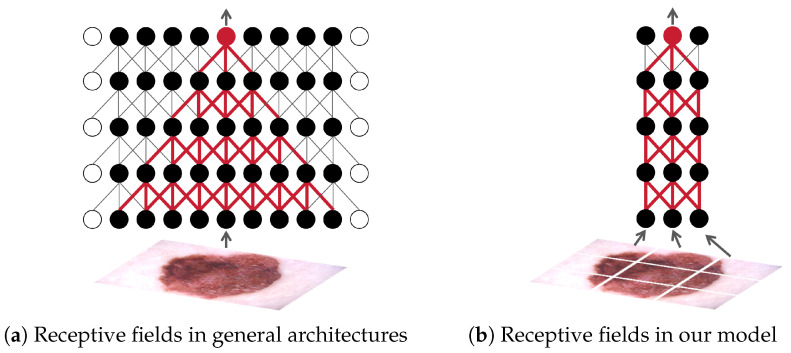
A simplified illustration of the receptive fields [22] of general convolutional neural networks (CNNs) and our model. For ease of understanding, a feature map is simplified as a row of solid circles and shown. The hollow circles represent padding. Here we use a 3 × 3 convolution with a stride of one as an example. In conventional CNNs, stacking multiple convolutional layers progressively enlarges the receptive field [22,23], allowing each pixel in the deep feature map to theoretically correspond to any location in the entire image. In contrast, our model divides the input image into independent patches, each processed by a network with a small receptive field and shared weights. The predictions (logits) from all patches are then averaged to produce the final output. This ensures that each pixel in the deep feature map strictly corresponds to a specific patch. Note that for illustration, we show a 3 × 3 grid of patches in this figure. In our actual work, a 225 × 225 input image is divided into 25 × 25 patches using a 33 × 33 window with a stride of eight, enabling more precise heatmap generation.

Specifically, as shown in Figure 1, in ECT-BoFM, a backbone network with a small receptive field extracts local features from small patches obtained by splitting an input image. The local features are then averaged to be the global descriptor for classification. In this way, each deep feature element strictly corresponds spatially to a particular patch of the input image. ECT-BoFM is trained with multiple tasks, including classification and the reconstruction of traditional EI and CTI features using deep features. The latter task not only enables the model to learn EI and CTI from the traditional features but also provides heatmaps from different perspectives. This allows us to understand not only where the model deems important but also the perspective (EI or CTI) upon which the model bases its importance assessment. Our contributions are as follows:

(i) We propose the novel ECT-BoFM for skin lesion diagnosis, which limits the size of the deep features’ receptive field to obtain precise heatmaps and learns CTI and EI from traditional features.
(ii) We propose a non-rigid reconstruction strategy that allows the model to learn from traditional features without being constrained by them.
(iii) ECT-BoFM, while sacrificing some capability in capturing global image information, significantly enhances the extraction of fine-grained features, which are more critical for skin lesion diagnosis, achieving high diagnostic performance. It surpassed recent state-of-the-art methods on two widely used public datasets, ISIC2018 [19] and ISIC2019 [19,20].
(iv) ECT-BoFM yields precise and detailed heatmaps showing the prediction basis, potentially making clinical applications of computer-aided diagnosis more trustworthy.
(v) Our experiments show that using only a small subset of image regions selected by our algorithm enables global-feature-learning models such as Vision Transformers [21] to achieve classification performance comparable to full-image inputs. This raises questions about the necessity and effectiveness of global learning mechanisms like self-attention and deep-stacked CNNs for skin disease diagnosis. We believe that this insight could inspire the development of more efficient network architectures in the future.

## 2. Related Work

Over the past decade, the rapid advancement of artificial intelligence has significantly transformed skin disease image diagnosis algorithms. Traditional methods, predominantly based on statistical principles and handcrafted feature extraction, have given way to deep learning-based approaches [17]. Traditional algorithms often relied on the integration of clinical medical knowledge with statistical techniques. Medical insights guided feature selection, focusing on characteristics such as shape, color, and texture, which have clear clinical interpretations. For example, malignant tumors like melanoma typically exhibit irregular edges and uneven pigmentation, making these features crucial for classification [24]. Statistical methods quantified these features, providing input for machine learning models, while medical expertise helped prioritize relevant features to enhance diagnostic accuracy.

In recent years, deep learning models have revolutionized the field, consistently outperforming traditional methods. State-of-the-art (SOTA) algorithms in skin disease diagnosis are predominantly deep learning-based, with innovations broadly categorized into advancements in network architecture [25,26,27] and training strategies [1,25,28].

### 2.1. Innovations in Network Architecture

Advancements in network architecture often involve designing models tailored to the specific characteristics of skin disease images or broader medical imaging tasks. For instance, Huo et al. [25] proposed HiFuse, a three-branch hierarchical network integrating convolution-based feature extraction, adaptive hierarchical feature fusion (HFF) blocks, and inverted residual MLPs. This model achieves outstanding performance on various medical image classification datasets, combining enhanced accuracy with computational efficiency.

More recently, with the emergence of novel token-based architectures like Vision Transformers [21], researchers have explored hybrid models that combine these token-based architectures with convolutional neural networks (CNNs) to further enhance skin disease classification. For example, Manzari et al. [26] introduced a robust CNN–Transformer hybrid model for medical image classification. By leveraging an efficient convolutional attention mechanism, the model captures long-range dependencies while reducing the quadratic complexity of self-attention. Additionally, the model emphasizes global structural features and utilizes feature space augmentation with mean and variance interpolation, achieving smoother decision boundaries and better generalization across diverse medical domains. Yue et al. [27] proposed MedMamba, a model that combines convolutional layers with state-space models (SSMs), effectively capturing both short- and long-range dependencies. By employing grouped convolution and channel-shuffle strategies, this approach balances computational efficiency with parameter reduction.

### 2.2. Innovations in Training Strategies

Innovations in training strategies have primarily targeted the imbalanced data distribution inherent in skin disease datasets. Certain diseases, such as melanoma, are relatively common, while others, such as actinic keratosis, are rare, resulting in a long-tailed distribution. Addressing this imbalance is critical to improving model performance. For instance, Gessert et al. [29] proposed a patch-based attention mechanism combined with pretrained architectures to enhance skin lesion classification. This method captures the global context across local patches, outperforming traditional aggregation approaches. Furthermore, diagnosis-guided loss weighting was introduced to incorporate meta-information about diagnostic methods, mitigating the impact of class imbalance. Zhang et al. [1] proposed a class-enhancement contrastive learning (ECL) strategy to address data imbalance and diagnostic difficulty. By utilizing a hybrid-proxy model and a cycle update strategy, minority class representation was improved. The proposed losses also accounted for both data imbalance and diagnostic complexity, leading to superior performance on imbalanced dermoscopic datasets.

### 2.3. The Position of Our Work

Our work primarily focuses on innovations in training strategies combined with certain network architectural enhancements. We rethink traditional handcrafted features used in skin disease classification, noting their medical and statistical interpretability, as well as their focus on extracting statistical features from small image patches and aggregating them at the final stage to capture fine-grained details. While the performance of handcrafted features has fallen behind deep learning-based methods, we argue that the underlying principles of these traditional approaches remain valuable. By leveraging suitable deep network architectures and designing training strategies, we explore how to extract relevant features guided by domain knowledge embedded in traditional methods, aiming to enhance the interpretability of deep features. Building on this, we integrated the bag-of-feature model into skin disease classification and adapted it to align with our tailored training strategy. This approach yielded precise and interpretable heatmaps from different traditional feature perspectives. Notably, despite not employing architectures capable of capturing global image information or training strategies specifically tailored for long-tailed challenges, our diagnostic performance surpasses the aforementioned SOTA methods. Additionally, we analyzed the impact of image patches selected by our model on classification results, comparing them to the influence of the entire image in deep learning. We believe that the findings from this analysis can provide valuable insights for the design of future dermatological lesion diagnostic models.

In addition to advances in architecture and training, substantial efforts have been made to improve the explainability of deep models in skin disease classification. Saliency-based visualization methods such as CAM [3], Grad-CAM [4], and their dermatology-specific adaptations [5,6,8] are widely adopted to highlight diagnostic regions. While effective to some extent, these methods often rely on deep-layer activations with large receptive fields, leading to heatmaps that may lack spatial precision or clinical specificity [9,30]. Some alternative techniques, including attribution-based methods such as SHAP [31], have also been explored.

Our work builds upon this line of research by proposing a local-feature-based framework that explicitly reconstructs traditional visual features, such as edge and color-texture, from deep features. This allows the model not only to localize informative regions with high spatial precision but also to generate clinically grounded interpretations aligned with expert diagnostic criteria (see details in Section 4.4).

## 3. Method

**Figure 2:**
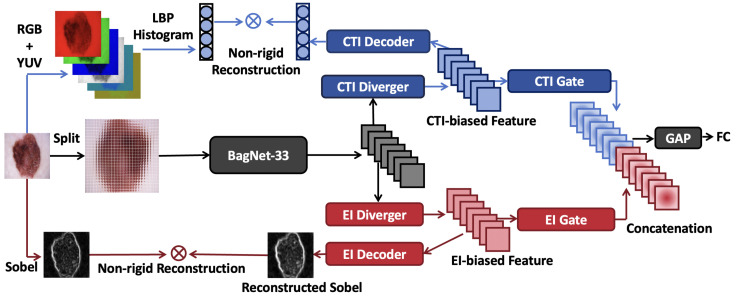
The overview of our edge- and color–texture-aware bag-of-local-features model.

Figure 2 illustrates the overall framework of ECT-BoFM. Let $I\in R^{H\times W\times 3}$ denote an input image. The framework conducts three main processes on *I*: (i) extracting the RGB (red, green, and blue) and YUV (luma, chrominance blue, and chrominance red) channels from *I* and concatenating the local binary pattern (LBP) histograms [32] (59-D) computed on the six channels to form a color–texture descriptor $C\in R^{354}$; (ii) obtaining a deep feature map *X* using a backbone network B; and (iii) deriving an EI map $E\in R^{H\times W}$ with the Sobel operator [15].

The backbone network B is based on BagNet-33 [18], so the receptive field is restricted to 33 × 33 pixels. In this paper, we set the stride for the 33 × 33 window as 8. The default input size of the original BagNet-33 model is 224 × 224, which results in the regions at the end of the input image not being traversed by the 33 × 33 window. To solve this problem, we set the size of *I* as 225 × 225, making $X\in R^{25\times 25\times 2048}$. Each element $x\in R^{1\times 1\times 2048}$ in *X* strictly corresponds to a certain 33 × 33 region in *I*.

Thereafter, a CTI diverger and an EI diverger take *X* as the input to generate a CTI-biased feature map $X_{\mathrm{CTI}}\in R^{25\times 25\times 2048}$ and an EI-biased feature map $X_{\mathrm{EI}}\in R^{25\times 25\times 2048}$, respectively. Then, a CTI decoder and an EI decoder take $X_{\mathrm{CTI}}$ and $X_{\mathrm{EI}}$ as the input to reconstruct *C* and *E*, respectively. During training, the decoders are optimized with the proposed non-rigid reconstruction strategy, which can prevent the optimization of the decoders from harming the classification performance. Meanwhile, a CTI gate and an EI gate enhance the features in $X_{\mathrm{CTI}}$ and $X_{\mathrm{EI}}$ that are beneficial for classification and weaken the unbeneficial features, respectively. The gated features are concatenated and then processed by global pooling (GAP) and fully connected (FC) layers for classification. During training, to obtain optimal results, we use a strategy called partial sharpness-aware minimization (PSAM) to optimize the whole framework. For inference, the decoders are removed, and the traditional features are not needed.

**Divergers:** The divergers are designed to diverge *X* into a CTI-biased feature map versus an EI-biased feature map. Let $f_{\mathrm{Conv}}^{M\times N\times H^{\prime }\times W^{\prime }}\left(.\right)$ denote a convolutional layer, which has *M* input channels, *N* output channels, and a kernel size of $H^{\prime }\times W^{\prime }$. All the convolutional layers given in this section do not use padding layers and have a stride of 1, so we omit them in the equations. The CTI and EI divergers have the same structure but do not share the parameters. The functions can be written as $X_{\mathrm{CTI}}=f_{\mathrm{Conv}}^{2048\times 2048\times 1\times 1}\left(X\right)$, $X_{\mathrm{EI}}=f_{\mathrm{Conv}}^{2048\times 2048\times 1\times 1}\left(X\right)$.

**Decoders:** The decoders are designed to reconstruct the color–texture descriptor $C\in R^{354}$ and edge map $E\in R^{225\times 225}$. This design allows the model to learn about CTI and EI during training and subsequently analyze the classification results from the perspectives of CTI and EI through visualization. In a typical reconstruction task, the decoder generates an output of the same size as the target and minimizes the point-to-point difference between this output and the target using a loss function such as the widely used Smooth L1 Loss. However, the main task in this paper is the classification task. Directly applying the typical strategies in the reconstruction task may result in degraded classification performance. For example, there may be competition between the low-level vision task (reconstruction) and high-level vision task (classification). Also, traditional feature extractors such as LBP and Sobel have been behind deep models in terms of classification performance for a long time. Making deep features strictly approximate traditional features may limit their usefulness. To solve this problem, we propose non-rigid reconstruction (NRR), which does not view the traditional features as direct reconstruction targets of $X_{\mathrm{CTI}}$ and $X_{\mathrm{EI}}$, but rather as a special case in distributions defined by $X_{\mathrm{CTI}}$ and $X_{\mathrm{EI}}$. Given $X_{\mathrm{CTI}}$ and $X_{\mathrm{EI}}$, NRR first defines two Gaussian distributions, $N(\mu_{\mathrm{CTI}},$$\sigma_{\mathrm{CTI}}^{2})$ and $N(\mu_{\mathrm{EI}},\;\sigma_{\mathrm{EI}}^{2})$, where $\mu_{\mathrm{CTI}},\;\sigma_{\mathrm{CTI}}\in R^{354}$ and $\mu_{\mathrm{EI}},\;\sigma_{\mathrm{EI}}\in R^{25\times 25\times 2048}$ are

(1) $$\begin{array}{cc} \; & \mu_{\mathrm{CTI}}=f_{\mathrm{FC}}^{128\times 354}(f_{\mathrm{ReLU}}(f_{\mathrm{FC}}^{2048\times 128}(f_{\mathrm{ReLU}}\left(f_{\mathrm{GAP}}\left(X_{\mathrm{CTI}}\right))))\right) \end{array}$$

(2) $$\begin{array}{cc} \; & \sigma_{\mathrm{CTI}}=f_{\mathrm{FC}}^{128\times 354}(f_{\mathrm{ReLU}}(f_{\mathrm{FC}}^{2048\times 128}(f_{\mathrm{ReLU}}\left(f_{\mathrm{GAP}}\left(X_{\mathrm{CTI}}\right))))\right) \end{array}$$

(3) $$\begin{array}{cc} \; & \mu_{\mathrm{EI}}=f_{\mathrm{Conv}}^{128\times 2048\times 1\times 1}\left(f_{\mathrm{ReLU}}\left(f_{\mathrm{Conv}}^{2048\times 128\times 1\times 1}\left(f_{\mathrm{ReLU}}\left(X_{\mathrm{EI}}\right)\right)\right)\right) \end{array}$$

(4) $$\begin{array}{cc} \; & \sigma_{\mathrm{EI}}=f_{\mathrm{Conv}}^{128\times 2048\times 1\times 1}\left(f_{\mathrm{ReLU}}\left(f_{\mathrm{Conv}}^{2048\times 128\times 1\times 1}\left(f_{\mathrm{ReLU}}\left(X_{\mathrm{EI}}\right)\right)\right)\right) \end{array}$$

Here, $f_{\mathrm{FC}}$, $f_{\mathrm{GAP}}$, and $f_{\mathrm{ReLU}}$ represent fully connected, global average pooling, and ReLU operations, respectively. Thereafter, we generate new features $X_{\mathrm{CTI}}^{\prime }\in R^{354}$ and $X_{\mathrm{EI}}^{\prime }\in R^{25\times 25\times 2048}$ by randomly sampling from the obtained distributions. However, due to the non-differentiability of random sampling, we follow Kingma et al. [33] to utilize reparameterization in practice. Let N(μ,$\sigma^{2})$ denote one of the above distributions, with the feature dimensionality omitted. Instead of directly sampling from N(μ,$\sigma^{2})$, the reparameterization trick samples ϵ from a standard normal distribution N(1,0) and computes the desired sample *z* as z=μ+σϵ. *z* can refer to either $X_{\mathrm{CTI}}^{\prime }$ or $X_{\mathrm{EI}}^{\prime }$). $X_{\mathrm{EI}}^{\prime }$ is then up-sampled as

(5) $$\begin{array}{cc} X_{\mathrm{EI}}^{\prime \prime } & =f_{\mathrm{Tconv}}^{2048\times 1\times 33\times 33}\left(f_{\mathrm{Conv}}^{128\times 2048\times 1\times 1}\left(f_{\mathrm{ReLU}}\left(f_{\mathrm{Conv}}^{2048\times 128\times 1\times 1}\left(f_{\mathrm{ReLU}}\left(X_{\mathrm{EI}}^{\prime }\right)\right)\right)\right)\right) \end{array}$$

where $f_{\mathrm{Tconv}}^{2048\times 1\times 33\times 33}\left(.\right)$ is a transposed convolution layer, and its stride is set as 8. Thus, the up-sampling process of this layer is in strict spatial correspondence with the down-sampling process of B. The proposed reconstruction loss is

(6) $$\begin{array}{c} L_{\mathrm{Rec}}=\log\left(L_{\mathrm{KL}}\left(C,X_{\mathrm{CTI}}^{\prime }\right)+1\right)+\log\left(L_{\mathrm{KL}}\left(E,X_{\mathrm{EI}}^{\prime \prime }\right)+1\right) \end{array}$$

where $L_{\mathrm{KL}}\left(.\right)$ denotes a KL loss with a sum reduction, and the log function can prevent the loss value from being too large. The design of NRR avoids strictly forcing $X_{\mathrm{CTI}}$ and $X_{\mathrm{CTI}}$ to adhere to traditional features to the extent that it may limit classification performance. At the same time, this design enables $X_{\mathrm{CTI}}$ and $X_{\mathrm{EI}}$ to learn macroscopic knowledge about CTI and EI during training that goes beyond the information covered by the traditional features [33].

**Gates:** The gates are designed to enhance features in $X_{\mathrm{CTI}}$ and $X_{\mathrm{EI}}$ that are beneficial for classification and weaken those that are unhelpful. The gated features $X_{\mathrm{CTI}}^{g}\in R^{25\times 25\times 2048}$ and $X_{\mathrm{EI}}^{g}\in R^{25\times 25\times 2048}$ are obtained as

(7) $$\begin{array}{cc} \; & X_{\mathrm{CTI}}^{g}=X_{\mathrm{CTI}}⊗A_{\mathrm{CTI}},\\ \; & \mathrm{where}\;A_{\mathrm{CTI}}=\mathrm{Sigmoid}\left(f_{\mathrm{Conv}}^{64\times 1\times 1\times 1}\left(f_{\mathrm{Conv}}^{2048\times 64\times 1\times 1}\left(X_{\mathrm{CTI}}\right)\right)\right) \end{array}$$

(8) $$\begin{array}{cc} \; & X_{\mathrm{EI}}^{g}=X_{\mathrm{EI}}⊗A_{\mathrm{EI}},\\ \; & \mathrm{where}\;A_{\mathrm{EI}}=\mathrm{Sigmoid}\left(f_{\mathrm{Conv}}^{64\times 1\times 1\times 1}\left(f_{\mathrm{Conv}}^{2048\times 64\times 1\times 1}\left(X_{\mathrm{EI}}\right)\right)\right) \end{array}$$

Here, ⊗ denotes the multiplication broadcast across the feature channels. $A_{\mathrm{CTI}}$ and $A_{\mathrm{EI}}$ are CTI and EI gating weights, respectively. $X_{\mathrm{CTI}}^{g}$ and $X_{\mathrm{EI}}^{g}$ then undergo concatenation, GAP, and FC operations to obtain categorical logits *l*.

**Partial Sharpness-aware Minimization:** During training, the overall loss can be written as $L=L_{S}\left(l,t\right)+L_{\mathrm{Rec}}$, where $L_{S}\left(.\right)$ denotes a softmax loss, and *l*, *t* are the predicted logits and target, respectively. Minimizing L is multi-task learning that encompasses both high-level and low-level vision tasks. To avoid pulling between tasks leading to suboptimal results, we propose PSAM, which is adapted from sharpness-aware minimization (SAM) [34]. Generally, the optimization of a model is defined as looking for the weights *w* that can result in the minimal loss such as $\min_{w}\;L_{w}+{\lambda ||w||}_{2}^{2}$, where ${\lambda ||w||}_{2}^{2}$ is an L2 regularization term. Unlike this general optimization strategy, SAM seeks parameters that lie in neighborhoods that have a uniformly low loss such as $\min_{w}\;\mathrm{SAM}\left(w\right)+{\lambda ||w||}_{2}^{2}$, where $\mathrm{SAM}\left(w\right)=\max_{{\left||\zeta |\right|}_{2}\leq \rho }\;L_{w+\zeta }$, and ρ>0 is a hyperparameter that defines the range of parameter neighborhoods that need to be searched. Instead of simply finding parameters leading to a low loss, SAM aims to find parameters where the entire neighborhood has a uniformly low training loss. This prevents the model from converging to sharp minima, where a parameter may lead to a low loss, but its neighboring parameters lead to a high loss. However, SAM may ignore some parameters that are in the sharp neighborhood but can actually lead to good performance. Thus, to further improve the results, we propose PSAM, which randomly selects 50% of the parameters to be updated with the general optimization strategy and the remaining 50% with SAM. PSAM also functions similarly to dropout by introducing noise as a form of regularization.

**Visualization:** In ECT-BoFM, each deep feature is in strict correspondence with a certain 33 × 33 patch in the input image. Given a 33 × 33 patch *p*, the model infers one logit $l_{p}^{k}$ for the predicted skin lesion class *k*, together with one EI gating weight $A_{\mathrm{EI}}^{p}$ and one CTI gating weight $A_{\mathrm{CTI}}^{p}$ (refer to Equations (7) and (8)). Thus, for each pixel of the heatmaps, we can obtain three types of values: (i) $A_{\mathrm{CTI}}^{p}l_{p}^{k}$, which denotes the model’s judgment of *p*’s importance from a CTI perspective; (ii) $A_{\mathrm{EI}}^{p}l_{p}^{k}$, which denotes the model’s judgment of *p*’s importance from an EI perspective; and (iii) $\alpha_{\mathrm{CTI}}A_{\mathrm{CTI}}^{p}l_{p}^{k}+\alpha_{\mathrm{EI}}A_{\mathrm{EI}}^{p}l_{p}^{k}$, which denotes the model’s overall judgment of *p*’s importance. Here, $\alpha_{\mathrm{CTI}}=\Sigma \left(w_{\mathrm{CTI}}^{\mathrm{FC}}\right)/\Sigma \left(w^{\mathrm{FC}}\right)$ and $\alpha_{\mathrm{EI}}=\Sigma \left(w_{\mathrm{EI}}^{\mathrm{FC}}\right)/\Sigma \left(w^{\mathrm{FC}}\right)$, where $w^{\mathrm{FC}}\in R^{4096}$ is the weights of the FC classifier for the predicted category *k*, and its negative values are set to 0. $w_{\mathrm{CTI}}^{\mathrm{FC}}\in R^{2048}$ and $w_{\mathrm{EI}}^{\mathrm{FC}}\in R^{2048}$ are the elements in $w^{\mathrm{FC}}$ handling the features from the CTI and EI gates, respectively. To generate the heatmaps, we pad *I* by 16 pixels on all sides with zeros and extract a total of 225 × 225 patches using a 33 × 33 window with a stride of 1. Then, we can obtain three heatmaps: a CTI heatmap, an EI heatmap, and an overall heatmap. The heatmaps are normalized with min-max normalization.

## 4. Experiments

### 4.1. Datasets and Implementation Details

**Datasets:** We evaluated the ECT-BoFM on two public dermoscopic datasets: ISIC2018 [19] with 10,015 images across seven classes (melanoma, melanocytic nevus, basal cell carcinoma, actinic keratosis/Bowen’s disease, benign keratosis, dermatofibroma, and vascular lesion) and ISIC2019 [19,20] with 25,331 images across eight classes (melanoma, melanocytic nevus, basal cell carcinoma, actinic keratosis, benign keratosis, dermatofibroma, vascular lesion, and squamous cell carcinoma).

**Implementation Details:** We trained the models using stochastic gradient descent (SGD) with 100 epochs, a momentum of 0.9, a weight decay of $5\times 10^{-4}$, and a mini-batch size of 16. The initial learning rate was 0.002, and it was updated with cosine annealing. During training, images are resized to 256×256 with random cropping and flipping as the only augmentations. For inference, we applied center cropping. We followed previous studies [1,35,36] to use three common evaluation metrics: accuracy (Acc), macro f1-score (F1), and macro area under the curve (AUC). We repeated the experiments five times and reported the average results.

### 4.2. Comparison with State-of-the-Art Methods

To show the superiority of ECT-BoFM, we compared the diagnostic performance of ECT-BoFM with recent state-of-the-art (SOTA) methods on ISIC 2018 and 2019. Different studies have used various partitions of the ISIC 2018 dataset to evaluate performance. In our experiments, we followed the partitions used in previous studies [1,35,36,37] and compared our results against those obtained with the same partitions. Specifically, there were three partitions: (i) train/val/test = 1:1:1; (ii) train/val/test = 3:1:1; and (iii) using all 10,015 images as training or validating samples and testing the obtained model on the ISIC Task 3 validation set (193 images). For the third partition, we used 30% of the training data for validation. The previous SOTA methods on ISIC 2019 predominantly split this dataset into train/val/test = 3:1:1 [1], and thus we followed this partition. The comparison results are shown in Table 1, Table 2, Table 3 and Table 4. On ISIC 2018, ECT-BoFM significantly outperformed other SOTA methods in all metrics. Overall, the closest competitor in performance to our method is CEM [28]. However, our approach not only outperforms CEM significantly across all diagnostic metrics but also eliminates the need of manually labeling concept tags on large datasets and conducting additional training for generalization to other datasets, as required by CEM. On ISIC 2019, ECT-BoFM performed marginally lower than the previous best method, ECL [1], in the F1 score but higher than all previous SOTA methods in the other two metrics. ECT-BoFM has a significant advantage with the highest level in most metrics.

Although ECT-BoFM achieves significant improvements over prior methods on ISIC2018, the performance gain on ISIC2019 is relatively limited. We attribute this to the increased number of diagnostic categories in ISIC2019, which introduces greater inter-class similarity and more complex decision boundaries. For example, actinic keratosis (AK), a precancerous condition, often shares visual characteristics with both benign keratosis (BKL) and squamous cell carcinoma (SCC), making it particularly difficult to distinguish. This visual ambiguity increases the challenge not just for our method, but for all competing approaches.

This trend is clearly reflected in our experimental results, as shown in Table 3 and Table 4; classification performance across all methods is consistently lower on ISIC2019 than on ISIC2018. Therefore, we believe the relatively smaller margin between our method and other state-of-the-art models on ISIC2019 does not reflect a specific limitation of our approach, but rather highlights a broader challenge faced by current techniques when dealing with fine-grained, high-confusion medical categories. These findings suggest that further methodological advances are still needed to address the inherent difficulty of multi-class skin lesion diagnosis in more diverse datasets.

### 4.3. Ablation Studies

The design of ECT-BoFM included a BagNet-based backbone B, a CTI stream, an EI stream, the non-rigid reconstruction strategy, and the PSAM training strategy. We conducted ablation studies to evaluate the components of this design. Additionally, to demonstrate the effectiveness of limiting receptive fields, we also constructed two backbones with unlimited receptive fields by inserting MlpFusion and TransFusion layers into the final two stages of the original backbone. These fusion layers reshape the input tensor for processing through MLP or Transformer layers designed to globally integrate the local features and then reshape back the output to maintain the original tensor dimensions. In these ablation experiments, we constructed a very challenging diagnosis task by randomly dividing ISIC-2018 into the training, validation, and test sets in a 1:1:1 ratio. The results are shown in Table 5.

Adding a single CTI or EI stream to B improved all three metrics, with the addition of both CTI and EI streams leading to a greater improvement in all three metrics compared to the original B and to B with the addition of a single CTI or EI stream. This result indicates that CTI and EI streams are complementary, and a fusion of them is beneficial for diagnostic performance. Whether using TransFusion or MlpFusion, the network’s performance decreases when the receptive field is not limited. This indicates that constraining the receptive field can enhance diagnostic performance. Then, when we trained B with the addition of the two streams without NRR, the accuracy and F1 score were even lower than those of the original B, indicating the effectiveness of NRR. Furthermore, introducing SAM decreased AUC and F1, while introducing PSAM improved all metrics.

### 4.4. Analysis of Interpretable Heatmaps

In this section, we analyze the fidelity of the generated interpretable heatmaps. All experimental results are based on a 3:1:1 train–val–test split of the ISIC 2018 dataset, with all relevant models trained using the SGD optimizer.

**Figure 3:**
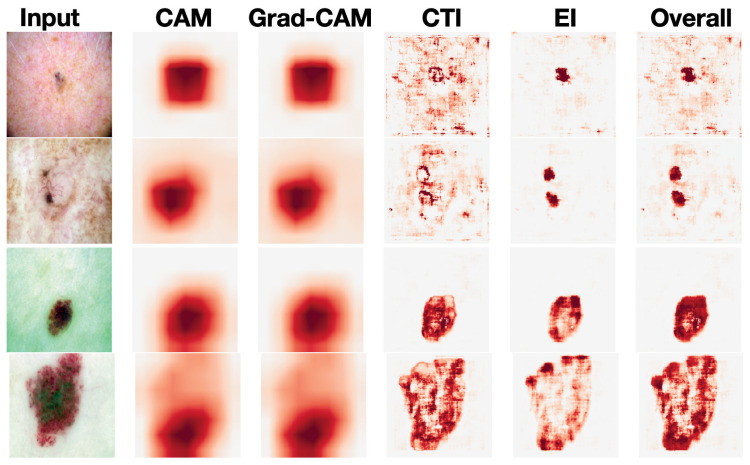
Comparison of visualization results.

**Visualization Results:** Figure 3 shows the visualization results of our model as well as those of Resnet50-based CAM and Grad-CAM, which are common strategies for prior interpretable dermatological diagnosis methods [3,4]. The CTI and EI heatmaps can provide explanations of the categorical predictions from two separate perspectives, enabling us to move a step forward in understanding the perspective from which the model makes its decisions. The overall heatmap can be seen as a synthesis of the CTI and EI heatmaps, capturing more comprehensive information. For dermatologic diagnosis, comprehensive information is beneficial. Consider the first row in Figure 3 as an example. The input image is a photo of a basal cell carcinoma (BCC). In this case, the EI stream mainly captures pigmented structures, such as a blue-gray ovoid nest, while the CTI stream mainly captures vascular structures, such as arborizing telangiectasias. Both are important for the diagnosis of BCC [17].

**Figure 4:**
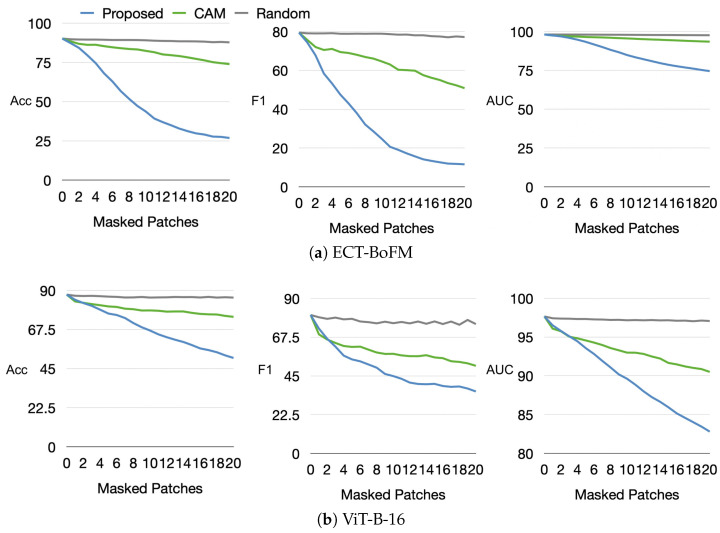
Decrease in classification performance in ECT-BoFM and ViT-B-16 if increasingly more patches are masked according to the heatmaps of ECT-BoFM, ResNet50+CAM, and random masking.

**Impact of Heatmap-Guided Masking on Accuracy.** To quantitatively analyze the fidelity of the generated ECT-BoFM heatmaps, we examined how accuracy changes when masking the most predictive 33 × 33 image patches. We employed three methods for masking patches: (i) defining the masking locations based on the ECT-BoFM heatmaps; (ii) randomly masking patches from all possible 33 × 33 windows with a stride of eight; and (iii) generating a heatmap using ResNet50 + CAM (note that CAM is similar to Grad-CAM for skin lesion diagnosis tasks, as illustrated in Figure 3; therefore we used CAM exclusively). For the third method, we traversed the generated heatmap with 33 × 33 windows and masked patches based on the highest sum of heatmap values within each window. We conducted these experiments on both the proposed ECT-BoFM and a trained ViT-B-16 model. The results, shown in Figure 4, demonstrate that masking the predictive patches identified by ECT-BoFM leads to a significant drop in diagnostic performance for both ECT-BoFM and ViT-B-16, compared to masking patches using other methods. Notably, masking just 20 patches, which constituted a small portion of the original image, resulted in negligible performance degradation when conducted randomly. In contrast, masking 20 important patches identified by ECT-BoFM caused a substantial performance decline in both models, which highlighted that the predictive patches provided by ECT-BoFM were naturally crucial to the model’s performance and were not only important to specific models but also fundamentally important.

As mentioned above, we used CAM exclusively based on its visual similarity to Grad-CAM in our experiments (Figure 3). To further support this choice, we note that dermoscopic images typically exhibit localized diagnostic features and relatively low spatial complexity, which naturally reduces the functional distinction between CAM- and gradient-based attribution methods. Previous studies in this domain, including Lucieri et al. [8] and Jiang et al. [6], have also observed that CAM and Grad-CAM tend to highlight nearly identical regions in skin lesion classification tasks. Hence, we adopted CAM throughout our framework to ensure simplicity and compatibility with our interpretable feature pathways, without compromising the quality or faithfulness of visual explanations.

**Results of Training with Heatmap-Identified Key Regions on Diagnostic Performance.**

**Figure 5:**
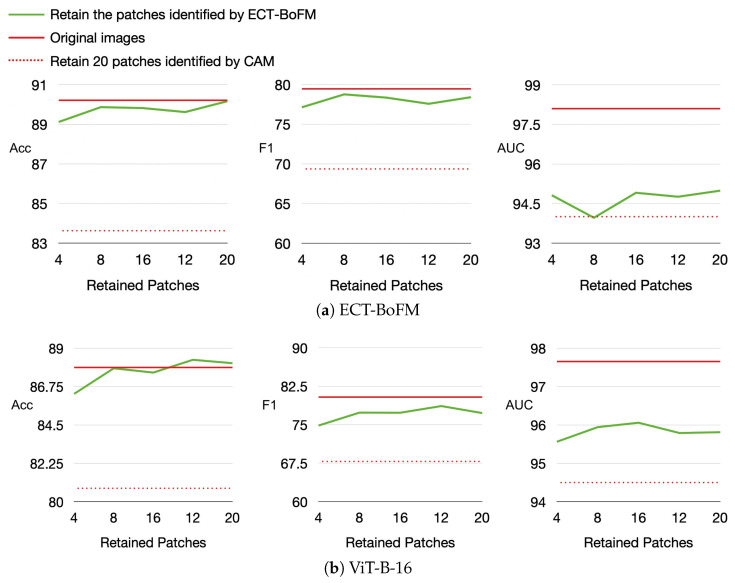
Classification performance on models trained with images where only a small portion of the patches is retained and the rest is masked.

We leveraged the interpretability of ECT-BoFM to analyze the impact of global and local information captured by models in skin lesion diagnosis. Using a trained ECT-BoFM, we identified the most predictive 33 × 33 image patches, retaining only these small regions while masking out the rest of the image. These modified images were then used to train both ViT-B-16 and ECT-BoFM. We conducted experiments where only 4, 8, 12, 16, or 20 of the most predictive image patches were retained, with the results shown in Figure 5. In Figure 5, we also provide two reference baselines: (i) the performance using original, unmasked images and (ii) the performance when retaining the 20 most predictive image patches identified by ResNet50+CAM from all possible 33 × 33 windows with a stride of eight while masking out the remaining regions.

As shown in Figure 5, whether training with ECT-BoFM or ViT-B-16, the diagnosis performance using the small number of patches identified by ECT-BoFM was significantly higher than that obtained using ResNet50+CAM. This indicated that the predictive patches identified by ECT-BoFM were genuinely critical regions, which is consistent with our earlier conclusions. Compared to the models trained on the original images, those using ECT-BoFM-identified patches achieved comparable or even superior accuracy, as well as similar F1 scores, but the AUC was better with models trained on the original images. These experimental results showed that for skin lesion diagnosis, a very small number of localized regions provide sufficient classification cues in most cases. While full input image information mainly enhanced the ability to differentiate some minority categories more comprehensively, the AUC improvement remained limited to about 2–3%. This phenomenon also applied to ViT models, which were designed to capture long-range dependencies between different local regions. Despite the extensive parameters and computations aimed at capturing such global information, the results in Figure 5 suggested that this design, while theoretically promising, might be less effective or even wasteful in practical skin lesion diagnosis. Training a ViT model with images where most regions were masked still achieved high diagnostic performance, which raises questions about whether ViT’s long-range dependency capture truly benefited skin lesion tasks. Future skin lesion diagnosis algorithms might be more effective by focusing on extracting local features and supplementing them with appropriate global information to improve the distinction of minority classes, thereby making better use of model parameters and computational resources.

### 4.5. Rethinking the Necessity of Global Modeling in Skin Lesion Diagnosis

In recent years, deep learning models with global modeling capabilities—such as Transformers with self-attention mechanisms—have shown impressive performance across various vision tasks. These models are based on the assumption that modeling long-range dependencies and holistic context is essential for high-level understanding, including in medical imaging. However, our findings suggest that this assumption may not universally hold for all medical imaging scenarios, particularly for skin lesion classification tasks.

As demonstrated in our experiments, local modeling alone—guided by interpretable and clinically relevant heatmaps—can achieve strong classification performance. For instance, Table 1 through Table 4 show that ECT-BoFM consistently outperforms or matches state-of-the-art methods on both ISIC2018 and ISIC2019 across multiple backbones, despite operating with a local receptive field and a limited global context. Figure 4 further shows that the removal of high-response regions, as highlighted by the interpretable channels, leads to substantial performance degradation, indicating that those localized regions carry essential diagnostic information. Figure 5 also reveals that even when only a small number of high-response patches are retained for training, classification performance remains close to that of the full model, further supporting the sufficiency of local features.

Architecturally, our use of BagNet-33 limits the effective receptive field to 33 × 33 pixels, minimizing the aggregation of non-local information. The model explicitly integrates edge and color–texture priors, which encourage it to focus on fine-grained features such as border irregularities and pigmentation details. The combination of these elements results in a lightweight, interpretable framework that is surprisingly effective without deep semantic fusion.

Nonetheless, we acknowledge that certain global visual patterns do carry diagnostic value in clinical practice. For example, dermatologists often assess asymmetry, lesion size relative to the surrounding area, or overall texture heterogeneity—features that cannot be captured by small receptive fields alone. Therefore, we do not claim that global information is irrelevant. Rather, our findings suggest that **existing generic global modeling mechanisms, such as standard self-attention layers, may not be well aligned with the domain-specific needs of skin lesion diagnosis**.

At the same time, although our method achieves state-of-the-art or competitive performance across multiple settings, there remains a gap to perfection. This opens a promising direction: **rethinking global modeling not by discarding it, but by designing new task-specific global mechanisms** that complement strong local representations with clinically meaningful global cues. Our findings offer a step in this direction by highlighting when and how local features dominate in this task and by raising the broader question of what kinds of global structures are truly needed in dermatological AI systems.

## 5. Conclusions

This paper introduces the novel ECT-BoFM for skin lesion diagnosis, which is required to perform two kinds of tasks: classification and reconstruction. The reconstruction task involves the model sampling new features from distributions defined by its deep features to complete the reconstruction of traditional edge and color–texture features. This approach allows the model to learn edge and color–texture information from traditional features effectively, without being overly constrained by them, thereby preventing negative effects on diagnostic performance. The proposed model obtained high diagnostic performance that clearly surpassed recent state-of-the-art methods on ISIC 2018 and ISIC 2019. By restricting the receptive field of deep features, this model generated precise and detailed heatmaps that reflect prediction basis from the edge, color–texture, and overall perspectives, potentially enhancing the reliability of AI-aided diagnosis. Analysis on ECT-BoFM’s interpretable heatmaps showed that masking a small number of the most predictive patches results in significant drops in diagnostic performance across models. Training with these patches alone achieved performance similar to using full images and suggested that, for other models designed to capture long-range dependencies and global information, the benefits from such capabilities are quite limited. We believe that the contribution of our work is not limited to proposing a new framework; it also provides valuable insights that can inspire the design of future dermatological lesion diagnostic models.

## Figures and Tables

**Table 1:** Comparison results on ISIC 2018 (train/val/test = 1:1:1).

	Acc	F1	AUC
ViT-B-16 (ICLR 2021 [21])	83.87	70.34	95.89
Vit-B-32 (ICLR 2021 [21])	85.70	74.57	96.67
MLP Mixer (NeurIPS 2021 [38])	80.25	61.06	93.76
EfficientViT B3 (ICCV 2023 [39])	75.70	47.21	92.43
MedViT (Elsevier CBM 2023 [26])	80.16	64.50	93.22
HiFuse (Elsevier BSPC 2024 [25])	82.77	70.71	95.84
MIL (CVPRW 2024 [40])	83.28	73.27	95.30
CEM (MICCAI 2024 [28])	86.07	75.15	96.72
MedMamba (arXiv 2024 [27])	77.34	53.81	94.72
Ours	**87.65**	**75.95**	**97.37**

**Table 2:** Comparison results on ISIC 2018 (test on the ISIC 2018 Task 3 validation set).

	Acc	F1	AUC
FCL (ICML 2019 [41])	82.85	39.87	72.14
ViT-B-16 (ICLR 2021 [21])	86.53	67.16	96.87
AdaptCL (ICCV 2021 [42])	83.17	37.64	70.71
DCLU (MICCAI 2023 [37])	85.10	40.58	73.57
MedViT (Elsevier CBM 2023 [26])	80.31	53.99	93.89
HiFuse (Elsevier BSPC 2024 [25])	83.94	73.37	97.49
MIL (CVPRW 2024 [40])	84.46	76.00	97.40
CEM (MICCAI 2024 [28])	86.53	68.60	97.18
MedMamba (arXiv 2024 [27])	79.27	56.59	92.64
Ours	**90.16**	**80.23**	**98.72**

**Table 3:** Comparison results on ISIC 2018 (train/val/test = 3:1:1).

	Acc	F1	AUC
ViT-B-16 (ICLR 2021 [21])	87.56	80.40	97.65
MWNL (IEEE TMI 2021 [43])	84.90	74.92	96.79
Hyb-SC (CVPR 2021 [44])	86.30	74.34	96.33
SCL (MICCAI 2021 [45])	86.13	74.27	96.56
BCL (CVPR 2022 [35])	84.92	71.57	95.61
TSC (CVPR 2022 [36])	85.94	74.94	95.83
ECL (MICCAI 2023 [1])	87.20	76.76	96.55
MedViT (Elsevier CBM 2023 [26])	82.78	67.75	96.04
HiFuse (Elsevier BSPC 2024 [25])	84.32	72.61	96.64
MIL (CVPRW 2024 [40])	84.82	72.52	96.90
CEM (MICCAI 2024 [28])	88.62	80.72	97.88
MedMamba (arXiv 2024 [27])	78.58	60.04	93.51
Ours	**91.01**	**83.25**	**98.33**

**Table 4:** Comparison results on ISIC 2019 (train/val/test = 3:1:1).

	Acc	F1	AUC
ViT-B-16 [21]	85.12	75.98	97.01
MWNL (IEEE TMI 2021 [43])	84.10	75.08	96.61
Hyb-SC (CVPR 2021 [44])	84.69	73.27	96.67
SCL (MICCAI [45])	84.60	75.07	96.21
BCL (CVPR 2022 [35])	83.47	73.50	95.95
TSC (CVPR 2022 [36])	84.75	75.13	95.84
ECL (MICCAI 2023 [1])	86.11	**79.46**	96.78
MedViT (Elsevier CBM 2023 [26])	79.89	71.19	95.51
HiFuse (Elsevier BSPC 2024 [25])	83.13	75.93	96.80
MIL (CVPRW 2024 [40])	83.07	74.44	96.82
CEM (MICCAI 2024 [28])	84.93	77.94	97.10
MedMamba (arXiv 2024 [27])	77.32	67.12	94.34
Ours	**86.23**	79.34	**97.58**

**Table 5:** Ablation studies on ISIC 2018 (train/val/test = 1:1:1).

	Only B	B + CTI	B + EI	B + EI + CTI	B + EI + CTI + TF	B + EI + CTI + MF	B + EI + CTI (w/o NRR)	B + EI + CTI + SAM	B + EI + CTI + PSAM
Acc	85.31	86.34	86.46	87.20	86.50	86.15	85.13	87.23	**87.65**
F1	72.63	73.87	73.17	75.62	74.84	73.57	71.67	75.43	**75.95**
AUC	96.69	97.06	96.47	97.32	96.90	96.54	97.09	97.30	**97.37**

‘w/o NRR’ indicates that the decoders were trained by smooth L1 loss, and the reconstruction results were generated directly from $X_{\mathrm{CTI}}$ and $X_{\mathrm{EI}}$ rather than samples from the distributions they define. ‘TF’ and ‘MF’ denote TransFusion and MlpFusion, respectively.

## Data Availability

The experimental results in this study are based on the publicly available ISIC 2018 and ISIC 2019 datasets, which can be accessed at https://challenge.isic-archive.com/data/#2018 (accessed on 22 July 2025) and https://challenge.isic-archive.com/data/#2019 (accessed on 22 July 2025), respectively. The source code and implementation details are publicly available at https://github.com/Dichao-Liu/ECT-BoFM (accessed on 22 July 2025) to support reproducibility.
